# Evaluation of Employment Loss and Financial Hardship Among US Adults With Disabilities During the COVID-19 Pandemic

**DOI:** 10.1001/jamanetworkopen.2023.3364

**Published:** 2023-03-16

**Authors:** Oliver T. Nguyen, Juhan Lee, Amir Alishahi Tabriz, Young-Rock Hong, Mohammed Al Jumayli, Maija Reblin, Kea Turner

**Affiliations:** 1 Department of Health Outcomes and Behavior, H. Lee Moffitt Cancer Center and Research Institute, Tampa, Florida; 2 Department of Psychiatry, Yale University, New Haven, Connecticut; 3 Department of Health Services Research, Management, and Policy, University of Florida, Gainesville; 4 Department of Senior Adult Oncology, H. Lee Moffitt Cancer Center and Research Institute, Tampa, Florida; 5 Department of Family Medicine, University of Vermont, Burlington

## Abstract

This cross-sectional study estimates the prevalence and determinants of employment loss and financial hardship among adults with disabilities during the COVID-19 pandemic.

## Introduction

As of 2021, 13.0% of US adults have at least 1 disability.^1^ People with disabilities often have more significant financial hardships due to fewer economic opportunities (eg, lower income).^2^ For instance, in February 2020, 31.1% of people with disabilities were employed compared with 74.8% of people without disabilities.^3^ Although negative financial consequences (eg, temporary or permanent employment loss) have been documented during the initial stages of COVID-19 among the general US adult population,^4^ less is known about potential disparities by disability type on job loss and financial hardship. To address this gap, we estimated the prevalence and determinants of employment loss and financial hardship among adults with disabilities during the COVID-19 pandemic.

## Methods

We used data from the US Census Bureau Household Pulse Survey from April 14, 2021, to March 31, 2022. The survey assessed information on US households’ employment loss and financial hardship during the COVID-19 pandemic and factors likely to affect finances. Disability type was defined as none, severe, or complete impairment in vision, hearing, cognition, or mobility or in multiple categories. Race and ethnicity were self-reported through the survey and included in the analyses given racial and ethnic disparities in employment loss during the pandemic.

Odds ratios (OR) and 95% CIs were calculated through a multivariable logistic regression model, adjusting for demographic factors, survey week, and COVID-19–related factors (eg, COVID-19 infection, teleworking). To develop nationally representative estimates, we used balanced repeated replication weights. Group differences in employment loss and financial hardship were determined using an equality of margins test. Two-sided *P* < .05 indicated statistical significance. All analyses were conducted with Stata, version 17.0 (StataCorp LLC).

## Results

Our weighted sample represented 223 796 313 adults (51.4% women and 48.6% men; 5.4% non-Hispanic Asian American, 16.9% Hispanic or Latino, 11.0 non-Hispanic Black or African American, 63.1% non-Hispanic White, and 3.6% other race or ethnicity [American Indian or Alaska Native, Chamorro, Pacific Islander, Samoan, and multiple races or ethnicities]; 10.9% with disability). Of these, 17.1% reported household employment loss in the past 4 weeks and 26.1% reported having difficulty with household financial expenses (Table 1). After controlling for other factors, respondents with vision disability (OR, 1.71 [95% CI, 1.57-1.86]) or multiple types of disabilities (OR, 1.97 [95% CI, 1.83-2.12]) were more likely to report employment loss compared with those with no disability (Table 2).

**Table 1.** Survey Respondent Characteristics

Characteristic	Survey respondents, No. (%)	
	Unweighted sample (n = 1 004 948)	Weighted population (n = 223 796 313)
Employment loss^a^		
Yes	121 687 (12.1)	38 267 743 (17.1)
No	883 251 (87.9)	185 528 569 (82.9)
Financial difficulty with expenses^b^		
A little or not at all difficult	804 910 (80.1)	165 304 756 (73.9)
Somewhat or very difficult	200 038 (19.9)	58 491 557 (26.1)
Disability status		
No disability	904 308 (90.0)	199 352 303 (89.1)
Visual disability	14 972 (1.5)	3 656 852 (1.6)
Hearing disability	11 134 (1.1)	2 285 093 (1.0)
Cognitive disability	27 252 (2.7)	7 077 144 (3.2)
Mobility disability	27 750 (2.8)	6 198 419 (2.8)
>1 Disability^c^	19 532 (1.9)	5 226 500 (2.3)
Sex		
Men	411 960 (41.0)	108 850 970 (48.6)
Women	592 988 (59.0)	114 945 343 (51.4)
Race and ethnicity		
Hispanic or Latino	92 747 (9.2)	37 872 607 (16.9)
Non-Hispanic Asian American	50 453 (5.0)	12 007 424 (5.4)
Non-Hispanic Black or African American	74 387 (7.4)	24 714 739 (11.0)
Non-Hispanic White	751 777 (74.8)	141 112 677 (63.1)
Other^d^	35 584 (3.5)	8 088 864 (3.6)
Age, y		
18-34	131 308 (13.1)	54 690 056 (24.4)
35-50	281 881 (28.0)	61 006 808 (27.3)
50-64	280 931 (28.0)	54 255 221 (24.2)
65-74	210 871 (21.0)	36 897 976 (16.5)
≥75	99 957 (9.9)	16 946 249 (7.6)
Children (aged <18 y)		
None	726 494 (72.3)	150 454 307 (67.2)
Child aged <5 y	21 374 (2.1)	6 208 147 (2.8)
Child aged 6-11 y	22 302 (2.2)	5 851 127 (2.6)
Child aged 12-17 y	40 974 (4.1)	11 184 837 (5.0)
>1 Child	193 804 (19.3)	50 097 893 (22.4)
Married		
Yes	578 405 (57.6)	124 442 975 (55.6)
No	426 543 (42.4)	99 353 338 (44.4)
Educational level		
Less than high school	19 637 (2.0)	17 311 326 (7.7)
High school graduate or equivalent	114 702 (11.4)	68 299 523 (30.5)
Some college	211 267 (21.0)	46 511 154 (20.8)
Associate’s degree	104 562 (10.4)	21 259 058 (9.5)
Bachelor’s degree	291 890 (29.0)	39 130 990 (17.5)
Graduate degree	262 890 (26.2)	31 284 259 (14.0)
Annual household income, $		
<25 000	85 490 (8.5)	24 080 743 (10.8)
25 000-34 999	69 446 (6.9)	18 462 230 (8.2)
35 000-49 999	85 078 (8.5)	20 147 787 (9.0)
50 000-74 999	136 682 (13.6)	28 694 987 (12.8)
75 000-99 999	114 827 (11.4)	21 673 534 (9.7)
≥100 000	307 139 (30.6)	50 373 143 (22.5)
Prefer not to say or did not report	206 286 (20.5)	60 363 885 (27.0)
Geographic region of US		
Northeast	158 580 (15.8)	38 230 964 (17.1)
South	317 747 (31.6)	85 464 447 (38.2)
Midwest	204 107 (20.3)	46 249 130 (20.7)
West	324 514 (32.3)	53 851 769 (24.1)
Remote work from home in the past week		
Yes	223 974 (22.3)	41 985 423 (18.8)
No	780 974 (77.7)	181 810 890 (81.2)
COVID-19 positive		
Yes	149 439 (14.9)	39 479 443 (17.6)
No	855 509 (85.1)	184 316 870 (82.4)
Survey week		
1 (April 14 to 26, 2021)	58 009 (5.8)	12 600 081 (5.6)
2 (April 28 to May 10, 2021)	66 032 (6.6)	12 777 285 (5.7)
3 (May 12 to 24, 2021)	61 387 (6.1)	12 746 221 (5.7)
4 (May 26 to June 7, 2021)	59 611 (5.9)	12 699 572 (5.7)
5 (June 9 to 21, 2021)	57 373 (5.7)	12 717 971 (5.7)
6 (June 23 to July 5, 2021)	55 859 (5.6)	12 647 333 (5.7)
7 (July 21 to August 2, 2021)	61 547 (6.1)	14 762 171 (6.6)
8 (August 4 to 16, 2021)	65 398 (6.5)	14 732 992 (6.6)
9 (August 18 to 30, 2021)	65 826 (6.6)	14 757 267 (6.6)
10 (September 1 to 13, 2021)	60 454 (6.0)	14 727 750 (6.6)
11 (September 15 to 27, 2021)	56 892 (5.7)	14 651 629 (6.5)
12 (September 29 to October 11, 2021)	54 196 (5.4)	14 648 848 (6.5)
13 (December 1 to 13, 2021)	57 932 (5.8)	14 816 604 (6.6)
14 (December 29, 2021, to January 10, 2022)	71 834 (7.1)	14 822 493 (6.6)
15 (January 26 to February 7, 2022)	72 111 (7.2)	14 747 744 (6.6)
16 (March 2 to 14, 2022)	80 487 (8.0)	14 940 346 (6.7)

^a^ Defined by the survey as personal or household employment loss in the past month and measured through the question “Have you, or has anyone in your household experienced a loss of employment income in the last 4 weeks?”

^b^ Defined as having a somewhat or very difficult time paying household expenses in the last 7 days and measured through the question “In the last 7 days, how difficult has it been for your household to pay for usual household expenses, including but not limited to food, rent or mortgage, car payments, medical expenses, student loans, and so on?”

^c^ Includes individuals with 2 or more disabilities, which was defined as severe or complete visual, hearing, mobility, or cognitive impairment.

^d^ Includes racial and ethnic groups, as defined by the survey instrument, that were underrepresented in the data set, including American Indian or Alaska Native, Chamorro, Pacific Islander, Samoan, and multiple races or ethnicities.

**Table 2.** Determinants of Employment Loss and Financial Hardship^a^

Characteristic	Model 1: employment loss^b^		Model 2: financial hardship^c^	
	OR (95% CI)	*P* value	OR (95% CI)	*P* value
Employment loss				
Yes	NA	NA	3.79 (3.68-3.91)	<.0001
No	NA	NA	1 [Reference]	NA
Disability status				
No disability	1 [Reference]	NA	1 [Reference]	NA
Visual disability	1.71 (1.57-1.86)	<.001	2.37 (2.18-2.57)	<.001
Hearing disability	1.42 (1.27-1.58)	<.001	1.90 (1.73-2.09)	<.001
Cognitive disability	1.61 (1.52-1.72)	<.001	2.57 (2.43-2.73)	<.001
Mobility disability	1.15 (1.07-1.24)	<.001	2.21 (2.08-2.35)	<.001
>1 Disability^d^	1.97 (1.83-2.12)	<.001	4.59 (4.24-4.97)	<.001
Sex				
Men	1 [Reference]	NA	1 [Reference]	NA
Women	0.84 (0.82-0.86)	<.001	1.14 (1.11-1.17)	<.001
Race and ethnicity				
Hispanic or Latino	1.73 (1.67-1.80)	<.001	1.14 (1.09-1.18)	<.001
Non-Hispanic Asian American	1.39 (1.32-1.47)	<.001	1.02 (0.96-1.08)	.41
Non-Hispanic Black or African American	1.60 (1.53-1.66)	<.001	1.49 (1.43-1.55)	<.001
Non-Hispanic White	1 [Reference]	NA	1 [Reference]	NA
Other^e^	1.60 (1.50-1.70)	<.001	1.42 (1.34-1.50)	<.001
Age category, y				
18-34	1 [Reference]	NA	1 [Reference]	NA
35-50	1.15 (1.11-1.20)	<.001	1.14 (1.10-1.18)	<.001
50-64	1.16 (1.11-1.21)	<.001	1.02 (0.98-1.05)	.26
65-74	0.62 (0.59-0.65)	<.001	0.62 (0.59-0.65)	<.001
≥75	0.40 (0.37-0.44)	<.001	0.43 (0.40-0.45)	<.001
Children (aged <18 y)				
None	1 [Reference]	NA	1 [Reference]	NA
Child aged <5 y	1.36 (1.25-1.49)	<.001	1.27 (1.18-1.37)	<.001
Child aged 6-11 y	1.25 (1.15-1.35)	<.001	1.29 (1.21-1.39)	<.001
Child aged 12-17 y	1.18 (1.11-1.26)	<.0001	1.23 (1.15-1.30)	<.001
>1 Child	1.38 (1.33-1.43)	<.001	1.45 (1.40-1.49)	<.001
Married				
Yes	0.88 (0.85-0.91)	<.001	0.82 (0.80-0.85)	<.001
No	1 [Reference]	NA	1 [Reference]	NA
Educational level				
Less than high school	1 [Reference]	NA	1 [Reference]	NA
High school graduate or equivalent	0.72 (0.67-0.77)	<.001	0.93 (0.87-0.99)	.02
Some college	0.70 (0.66-0.75)	<.001	0.94 (0.88-1.00)	.06
Associate’s degree	0.67 (0.63-0.72)	<.001	0.90 (0.85-0.97)	.005
Bachelor’s degree	0.54 (0.51-0.58)	<.001	0.65 (0.61-0.69)	<.001
Graduate degree	0.49 (0.46-0.52)	<.001	0.58 (0.54-0.62)	<.001
Annual household income, $				
<25 000	1 [Reference]	NA	1 [Reference]	NA
25 000-34 999	0.82 (0.77-0.86)	<.001	0.75 (0.72-0.79)	<.001
35 000-49 999	0.67 (0.63-0.71)	<.001	0.58 (0.55-0.61)	<.001
50 000-74 999	0.54 (0.51-0.57)	<.001	0.38 (0.37-0.40)	<.001
75 000-99 999	0.43 (0.40-0.45)	<.001	0.26 (0.25-0.28)	<.001
≥100 000	0.30 (0.28-0.32)	<.001	0.13 (0.12-0.14)	<.001
Prefer not to say or did not report	0.59 (0.56-0.62)	<.001	0.28 (0.27-0.29)	<.001
Geographic region				
Northeast	1 [Reference]	NA	1 [Reference]	NA
South	0.91 (0.88-0.95)	<.001	0.97 (0.94-1.01)	.26
Midwest	0.82 (0.78-0.85)	<.001	0.86 (0.83-0.89)	<.001
West	1.02 (0.98-1.07)	.24	0.92 (0.89-0.96)	<.001
Telework from home in the past week				
Yes	0.85 (0.82-0.89)	<.001	0.96 (0.92-0.99)	.02
No	1 [Reference]	NA	1 [Reference]	NA
COVID-19 positive				
Yes	1.42 (1.38-1.47)	<.001	1.06 (1.02-1.09)	<.001
No	1 [Reference]	NA	1 [Reference]	NA
Survey week^f^				
1 (April 14 to 26, 2021)	1 [Reference]	NA	1 [Reference]	NA
2 (April 28 to May 10, 2021)	0.98 (0.91-1.05)	.67	1.06 (0.99-1.14)	.07
3 (May 12 to 24, 2021)	0.94 (0.87-1.01)	.14	1.05 (0.98-1.13)	.14
4 (May 26 to June 7, 2021)	0.90 (0.84-0.97)	.009	1.11 (1.03-1.19)	.003
5 (June 9 to 21, 2021)	0.89 (0.83-0.96)	.004	1.21 (1.12-1.29)	<.001
6 (June 23 to July 5, 2021)	0.93 (0.86-1.01)	.09	1.11 (1.03-1.19)	.004
7 (July 21 to August 2, 2021)	0.89 (0.82-0.96)	.004	1.08 (1.00-1.16)	.02
8 (August 4 to 16, 2021)	0.81 (0.75-0.87)	<.001	1.08 (1.01-1.16)	.02
9 (August 18 to 30, 2021)	0.84 (0.78-0.91)	<.001	1.08 (1.01-1.16)	.02
10 (September 1 to 13, 2021)	0.88 (0.82-0.95)	.002	1.10 (1.03-1.18)	.004
11 (September 15 to 27, 2021)	0.86 (0.80-0.93)	<.001	1.15 (1.07-1.23)	<.001
12 (September 29 to October 11, 2021)	0.79 (0.73-0.85)	<.001	1.19 (1.11-1.28)	<.001
13 (December 1 to 13, 2021)	0.72 (0.66-0.78)	<.001	1.31 (1.21-1.41)	<.001
14 (December 29, 2021, to January 10, 2022)	0.83 (0.77-0.89)	<.001	1.33 (1.24-1.42)	<.001
15 (January 26 to February 7, 2022)	0.92 (0.86-0.99)	.03	1.36 (1.27-1.45)	<.001
16 (March 2 to 14, 2022)	0.59 (0.55-0.63)	<.001	1.66 (1.56-1.77)	<.001

Abbreviations: NA, not applicable; OR, odds ratio.

^a^ Includes 1 004 948 survey respondents in the unweighted sample and 223 796 313 in the weighted population.

^b^ Defined by the survey as personal or household employment loss in the past month and measured through the question “Have you, or has anyone in your household experienced a loss of employment income in the last 4 weeks?”

^c^ Defined as having a somewhat or very difficult time paying household expenses in the last 7 days and measured through the question “In the last 7 days, how difficult has it been for your household to pay for usual household expenses, including but not limited to food, rent or mortgage, car payments, medical expenses, student loans, and so on?”

^d^ Includes individuals with 2 or more disabilities, which was defined as severe or complete visual, hearing, mobility, or cognitive impairment.

^e^ Includes racial and ethnic groups, as defined by the survey instrument, that were underrepresented in the data set, including American Indian or Alaska Native, Chamorro, Pacific Islander, Samoan, and multiple races or ethnicities.

^f^ Response rates for the weekly surveys ranged from 5.4% to 7.9%.

Controlling for other factors, we found respondents reporting employment loss were more likely to report financial hardship (OR, 3.79 [95% CI, 3.68-3.91]) compared with those reporting no employment loss. In addition, respondents reporting cognitive disability (OR, 2.57 [95% CI, 2.43-2.73), visual disability (OR, 2.37 [95% CI, 2.18-2.57]), or multiple types of disabilities (OR, 4.59 [95% CI, 4.24-4.97]) were more likely to report financial hardship compared with those with no disability.

## Discussion

We found people with disabilities were more likely to report household employment loss and financial hardship during the initial COVID-19 pandemic, which are especially pronounced among racial and ethnic minority respondents. These findings suggest people with disabilities may be disproportionately affected by the initial pandemic and may require additional resources and policy strategies (eg, training programs, workplace accommodations)^5^ as several labor markets adapt to the pandemic (eg, shifting to remote working).

Study limitations include self-report bias on financial hardship and limited information on the reason for employment loss (eg, furlough vs layoff), receipt of economic stimulus payments, and length of unemployment experienced. Our findings may also be affected by regional-level variations and the survey’s definition of disability by type of activity limitations. Notwithstanding, this study highlights the prevalence of employment loss and financial hardship during the early stages of the COVID-19 pandemic, which can inform initiatives (eg, expanding adoption of assistive technologies)^6^ to assist people with disabilities to regain and maintain meaningful employment as one mechanism to reduce financial hardship among this population.

## Notes

Supplement.
Data Sharing Statement
