# Supplementary material for: Evaluation of Employment Loss and Financial Hardship Among US Adults With Disabilities During the COVID-19 Pandemic
Source: JAMA Netw Open. 2023 Mar 16;6(3):e233364. doi: 10.1001/jamanetworkopen.2023.3364 (PMC10020877; doi:10.1001/jamanetworkopen.2023.3364)
Supplement: Supplement. — Data Sharing Statement [file jamanetwopen-e233364-s001.pdf]

Data Sharing Statement

Nguyen. Evaluation of Employment Loss and Financial Hardship Among US Adults With Disabilities During the COVID-19 Pandemic. *JAMA Netw Open*. Published March 16, 2023. doi:10.1001/jamanetworkopen.2023.3364

Data
Data available: No

Additional Information
Explanation for why data not available: Data available upon reasonable request to the corresponding author.
